# Using Social Media to Recruit a Diverse Sample of Participants for a Mobile Health (mHealth) Intervention to Increase Physical Activity: Exploratory Study

**DOI:** 10.2196/56329

**Published:** 2025-04-28

**Authors:** Laura Pathak, Rosa Hernandez-Ramos, Karina Rosales, Jose Miramontes-Gomez, Faviola Garcia, Vivian Yip, Suchitra Sudarshan, Anupama Gunshekar Cemballi, Courtney Lyles, Adrian Aguilera

**Affiliations:** 1 School of Social Welfare, University of California, Berkeley, 120 Haviland Hall, Berkeley, CA, 94720, United States, 1 510-703-5496; 2 Department of Psychological Science, University of California, Irvine, Irvine, CA, United States; 3 Department of the History of Medicine, Johns Hopkins University, Baltimore, MD, United States; 4 Whole Person Integrated Care, San Francisco Department of Public Health, San Francisco, CA, United States; 5 Department of Molecular and Cell Biology, University of California, Berkeley, Berkeley, CA, United States; 6 Center for Healthcare Research and Policy, University of California Davis School of Medicine, Davis, CA, United States; 7 Department of Psychiatry, University of California, San Francisco, San Francisco, CA, United States

**Keywords:** mHealth, social media, research subject recruitment, Spanish speakers, Spanish, physical activity, clinical research, social media platform, smartphone, text messaging, machine learning, diabetes, depression, Facebook, depressive symptoms, screening survey, online platform, cost-effectiveness, mobile health

## Abstract

**Background:**

Recruitment of demographically diverse samples in clinical research is often challenging and even more so during the COVID-19 pandemic when traditional in-person recruitment methods could not be implemented. Social media platforms offer an alternative approach for recruiting diverse samples of participants for clinical trials, including those testing digital health interventions. This approach allowed for a quicker recruitment process without the physical constraints associated with traditional in-person methods.

**Objective:**

This study aimed to detail the online and social media campaigns used to recruit participants for “Diabetes and Mental Health Adaptive Notification Tracking and Evaluation” (DIAMANTE), a randomized controlled trial testing a smartphone-based intervention (a text messaging system that uses machine learning to personalize content) to increase physical activity for patients with diabetes and depression. In describing the recruitment process, we seek to offer insights to the research community on recruitment through online and social media advertisements for diverse communities.

**Methods:**

This study sought to recruit demographically diverse individuals in the United States through social media, including paid advertisements on Craigslist and Facebook (Meta). For the DIAMANTE project recruitment, we created 18 personas that mapped into the population’s target demographics using a user-centered design methodology. We deployed targeted English and Spanish ads on Craigslist and Facebook in 78 cities based on county-level demographics and diabetes prevalence data to target diverse individuals aged 18‐75 years old, who had been diagnosed with diabetes and had documented depressive symptoms.

**Results:**

A total of 1379 individuals completed the study’s initial screening survey. Of those, 71 respondents on Facebook and 508 on Craigslist were interested in enrolling in our study. In total, 26 out of 58 (45%) eligible respondents from Facebook and 50 out of 235 (21.3%) eligible respondents from Craigslist were eventually recruited in the randomized controlled trial. In all, both platforms showed poor performance in recruiting Spanish speakers, with Facebook advertisements accounting for 0 and Craigslist for 4 (5.3%) of such participants. When it came to English speakers, Craigslist proved to be the better performing platform compared to Facebook, both in terms of reach (579 vs 71) and cost-effectiveness (US $67.61 in average cost per recruited participant vs US $80.16). While Craigslist ads reached more people, resulting in more completed screening surveys than Facebook ads, there was a higher number of ineligible and incomplete enrollment from Craigslist compared with Facebook, leading to a relatively lower conversion rate (9.4% vs 36.6%). Importantly, participants recruited through Craigslist were more ethnically and racially diverse than those recruited from Facebook.

**Conclusions:**

Results from this study revealed that it is possible to recruit diverse sample sets using social media and online advertisements. However, despite targeted recruitment efforts, social media recruitment of Spanish speakers proved especially challenging and costly. Further research is needed to determine systematic, online methods for recruiting marginalized communities.

## Introduction

### Health Disparities and mHealth

Research on the use of information technologies, particularly mobile health (mHealth), as a service delivery paradigm for health care systems has proliferated in the last decade due to the potential of these tools to help bridge gaps in care for underserved individuals [1,2]. Coupled with early evidence supporting its efficacy [3-5], the undisputed ubiquity of mobile technology, and strong interest among patients [6], there is a clear potential for harnessing mHealth to widen health care access. However, marginalized communities, particularly African Americans and Latinos, are underrepresented in mHealth research [7,8], and there are known barriers to recruiting samples of historically underrepresented communities in studies [9]. As a result, there is limited science to support mHealth treatment recommendations for these communities, which arguably worsens existing racial and ethnic health disparities.

### Social Media Recruitment for mHealth Research

Recruitment challenges with hard-to-reach populations in mHealth clinical trials were aggravated in the wake of the COVID-19 pandemic when reliance on some traditional recruitment methods, such as using flyers, word-of-mouth, or in-person recruitment by a health care provider, was either not feasible or fully implementable. Using social media platforms such as Facebook (Meta), Craigslist, Google Ad Words, Instagram (Meta), and Twitter (subsequently rebranded X) for recruitment offered a potentially faster, more affordable, and efficient alternative to traditional recruitment methods of national samples. Online recruitment was emphasized in multiple mHealth research areas such as depression [8,10-14], anxiety [10], smoking and tobacco use [15-17], diabetes [18], cancer survivor support [19], and healthy weight gain in pregnancy [20].

While there are a few promising studies leveraging social media to reach historically marginalized communities for surveys or broader outreach [10,21,22], the evidence on recruiting marginalized populations through social media for mHealth randomized controlled trials (RCTs) is more limited. Pratap et al [8] conducted a fully remotely run RCT testing the feasibility of using mental health apps to assess and treat moderate depressive symptoms among Latino adults. They found that it was substantially more expensive and labor-intensive to recruit Spanish-speaking participants through social media relative to English-speaking Latino and other groups, with participant acquisition costs differing dramatically between Spanish (US $31 per enrolled participant) and English speakers (US $1.49 per enrolled participant). Bunge et al [17] reported successful Facebook recruitment of Spanish-speaking smokers through Facebook in a clinical trial of a smoking cessation web app. However, the authors also described concerns about the high costs of recruiting such participants compared with English speakers. These data suggest that, though feasible, recruiting Spanish-speaking respondents for mHealth RCTs through social media can be expensive. Therefore, testing more targeted recruitment strategies on social media platforms has the potential to achieve better affordability.

Furthermore, there are also challenges when it comes to understanding effective social media advertising content for research recruitment. For example, although researchers have reported on Facebook ad development practices for health research recruitment [17,23], the platform’s frequently changing ad management interface, abstruse content posting rules, and proprietary algorithm make it challenging to standardize best practices for advertising content tailored to specific populations. However, an experimental study by Choi et al [24] found that recruitment rates, engagement, and participant characteristics varied as a function of changes in Facebook advertisement content. The authors thus emphasized the importance of the content of social media advertisements being carefully crafted for the target population [24]. The literature contains fewer reports regarding Craigslist ad design, possibly due to the platform’s less restricted options for content display, which resemble newspaper advertisements with a headline, body text, a map view, and optional picture(s).

Given the state of the evidence, it is clear that understanding the best methods for effective recruitment of hard-to-reach populations in mHealth studies is paramount to advancing digital health science. In this paper, we aim to detail the social media campaigns we used to recruit participants for “Diabetes and Mental Health Adaptive Notification Tracking and Evaluation” (DIAMANTE), an RCT testing a smartphone-based intervention (described below) to increase physical activity for individuals with diabetes and depression. Specifically, we aim to evaluate the performance of social media platforms for research recruitment and the associated costs by population and platform. In describing the process and reporting our recruitment results, we seek to offer the clinical research community insights on best practices for recruiting marginalized communities through social media advertisements.

## Methods

### Study Overview

The recruitment methods and data reported herein are part of the DIAMANTE RCT (NCT 03490253), which seeks to evaluate the effectiveness of a mobile app that uses reinforcement learning algorithms to predict which categories of health text-messages are most effective in increasing a participant’s physical activity based on previous physical activity data (number of steps) and other demographic and contextual variables [25]. Importantly, our recruitment methods were informed by lessons learned in using a similar recruitment approach in another study aiming to deliver a text-messaging program to individuals struggling with social distancing during the COVID-19 pandemic (StayWell at Home) [26].

This study sought to recruit individuals in the United States who were low-income, spoke English or Spanish, and were from diverse racial and ethnic backgrounds through paid advertisements on Craigslist and Facebook. We deployed targeted English and Spanish ads on Craigslist and Facebook in 78 cities based on county-level demographic and diabetes prevalence data (detailed below). Craigslist is a marketplace website used for posting and searching free and paid local classified ads in different categories, including housing rentals, job postings, for-sale items, and community events. Facebook is a social networking website used primarily for connecting with friends and colleagues through direct messaging and content sharing. We chose these 2 platforms due to their wide reach (Craigslist and Facebook have 250 million and 2.9 billion monthly active users, respectively) and based on the limited literature suggesting they were effective in recruiting diverse samples for digital health studies [8,17].

### Participants

Eligible participants for the DIAMANTE trial included individuals aged 18‐75 years who had been diagnosed with diabetes and had documented depressive symptoms (score>5 on the eight-item Patient Health Questionnaire depression scale [PHQ-8]). Before and early on in the COVID-19 pandemic, we had recruited 15 (5 English and 10 Spanish) participants for the trial using traditional methods (ie, health care provider referrals and flyers) in a large safety-net health care system located in the Western United States. Starting in the summer of 2020, we pivoted our approach to using online and social media platforms as a means to recruit DIAMANTE participants from the broader community due to shelter-in-place and social distancing protocols. We later expanded to a hybrid approach and used traditional (implemented remotely) and social media recruitment methods [27].

To ensure that social media recruits were similar to our clinical participants, the research team designed social media ads to target US residents with both type 2 diabetes and clinical depression or depressive symptoms, who were low-income, English or Spanish-speaking, and from diverse racial and ethnic backgrounds. We decided to focus on major metropolitan regions with higher rates of type 2 diabetes prevalence and higher concentrations of populations demographically similar to our clinical participants. We analyzed Centers for Disease Control and Prevention county-level data for type 2 diabetes prevalence throughout the United States and Census Bureau county-level demographic data to determine which metropolitan regions were most similar to the area in the Western US state where the DIAMANTE trial was originally set in. If a metropolitan region was not demographically similar to our study area, we placed a greater emphasis on regions with higher rates of type 2 diabetes prevalence [28].

### Development of Recruitment Ads

#### Facebook

**Figure 1.**
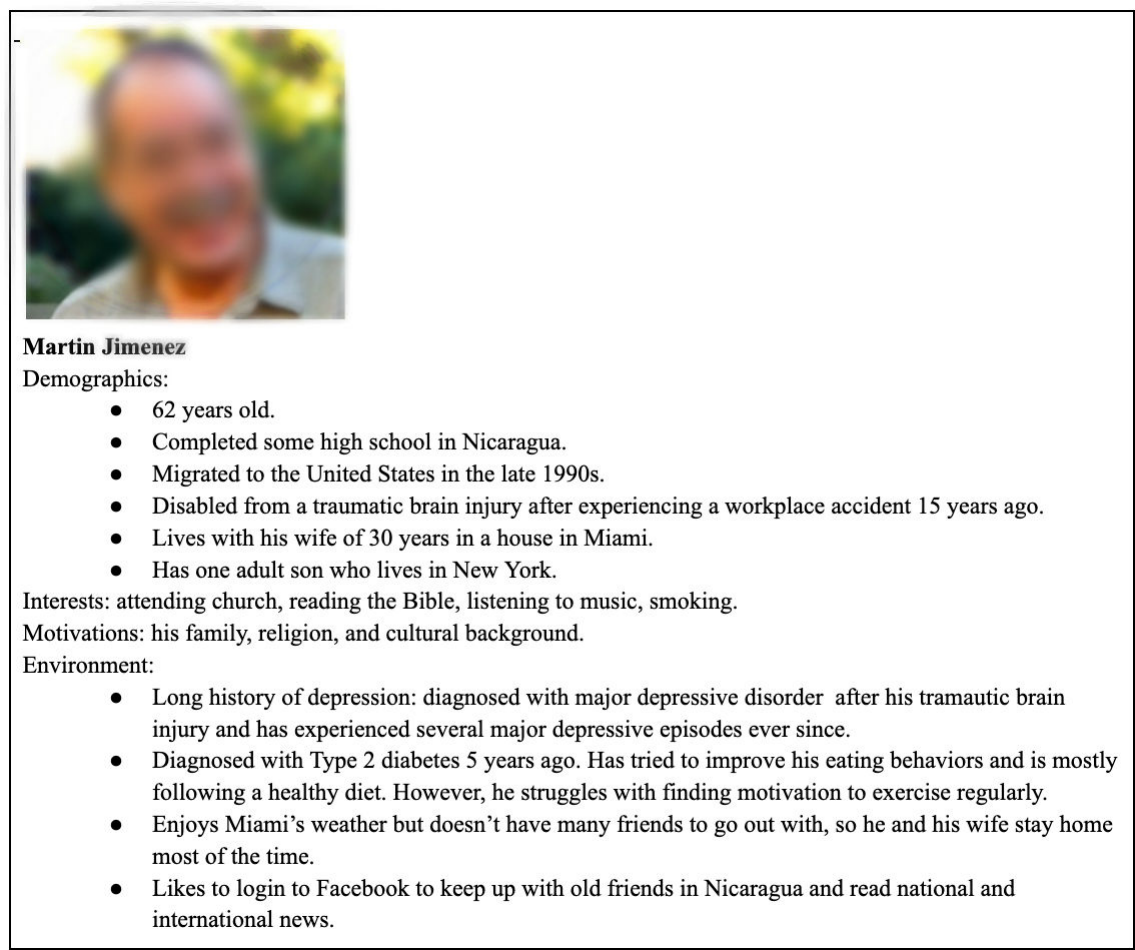
Sample persona of an older Latino man.

In June 2020, we created 18 personas that mapped into our target populations’ demographics, which comprised of different combinations of sex (male, female), language (Spanish, English), age (younger, older), and race or ethnicity (African American, White, Hispanic or Latino, Asian) categories. A persona is a fictional characterization of a user that includes specific characteristics and demographics found in the target population [29]. A design method commonly used under the User Centered Design framework and more broadly in the fields of human factors engineering and human-computer interaction, personas help think through the early design aspects of a new product that aims to be culturally appropriate, relevant, and user-friendly to the target user or user base [30]. To create the personas (refer to Figure 1 for an example), the research team harnessed personal and professional experiences working with our target populations and subpopulations to write up brief, fictional descriptions of demographic information, interests, motivations, and environmental factors. We paired the descriptions with stock pictures of people who embodied the physical characteristics of each persona.

In August 2020, we used the 18 personas as inspiration to craft 60 Facebook ads following the COM-B (Capability, Opportunity, Motivation and Behavior) model [31] as a framework for content development (refer to Multimedia Appendix 1 for a table of ad copy by persona). The COM-B model posits that behavior change entails an interacting system consisting of 3 constructs: capability, opportunity, and motivation. Effective behavior change occurs when one or more of these components are changed as it rearranges the system and minimizes the risk of default [30]. Hence, each persona had a corresponding set of 3 ads: an ad with a “capability” orientation, another with an “opportunity” orientation, and a third with a “motivation” orientation. The “capability” construct refers to the skills and knowledge necessary for change; in this case, we framed this subset of ads based on the positive physical and social outcomes of exercise. The “opportunity” construct taps into the socio environmental factors that enable change, so we created this subset of ads with cues for exercise opportunities or suggestions on minimizing barriers to exercise. Finally, as the “motivation” construct refers to the internal cognitive processes that direct behavior, this group of ads contained messaging to boost self-efficacy, including self-management skills and confidence to exercise.

**Figure 2.**
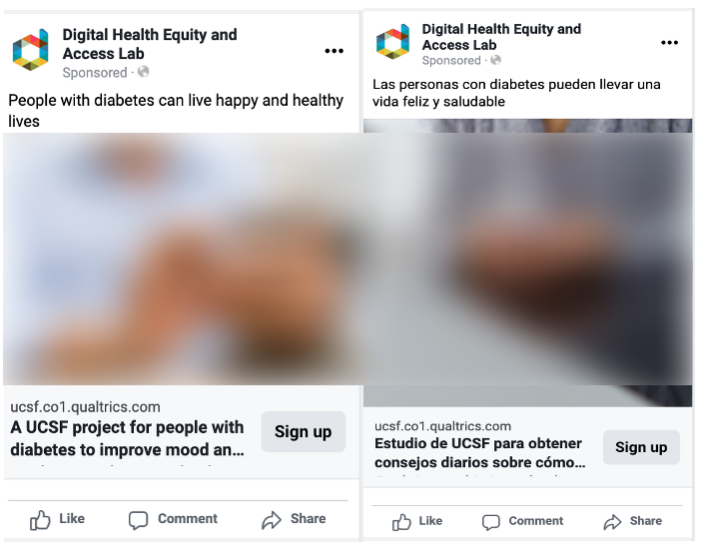
Example ads on Facebook. UCSF: University of California, San Francisco.

In addition, we created 6 broad ads (targeting a broad audience, not specific to any demographic group besides diabetes and depression diagnoses). Three of those 6 ads had vague wording around “well-being,” and 3 had more concrete wording on diabetes and depression (again, each type of wording adapted to the 3 COM-B theoretical constructs). Because Facebook requires that ads not contain “content that asserts or implies personal attributes” [32], we used indirect, as opposed to direct, phrasing (“people with diabetes” vs “do you have diabetes?”). Furthermore, we made references to “low mood” rather than “depression,” given the stigmatizing nature of this condition, and, based on findings in previous literature [17], framed the content to induce self-efficacy for behavior change, as opposed to fear. In general, the ad copy consisted of a brief sentence or phrase written in terms of the corresponding COM-B construct and persona, a brief study description, a link or button to take the screening survey, and a nondescript stock picture of a person holding a smartphone (refer to Figure 2 for Facebook ad examples).

Although we created the copy for 66 Facebook ads based on our 18 personas and general wording around health conditions or well-being, given budget constraints, we chose a subset of 7 ads for posting on Facebook (Multimedia Appendix 1). In this subset we chose to include some of the broad ads (4) in order to achieve high reach and some of the persona ads (3) that best encapsulated the demographic characteristics of the populations we were targeting (ie, older Latina females, older Latino males, older Black females, and older Black males). We then randomly selected the theoretical construct for each chosen ad category.

#### Craigslist

**Figure 3.**
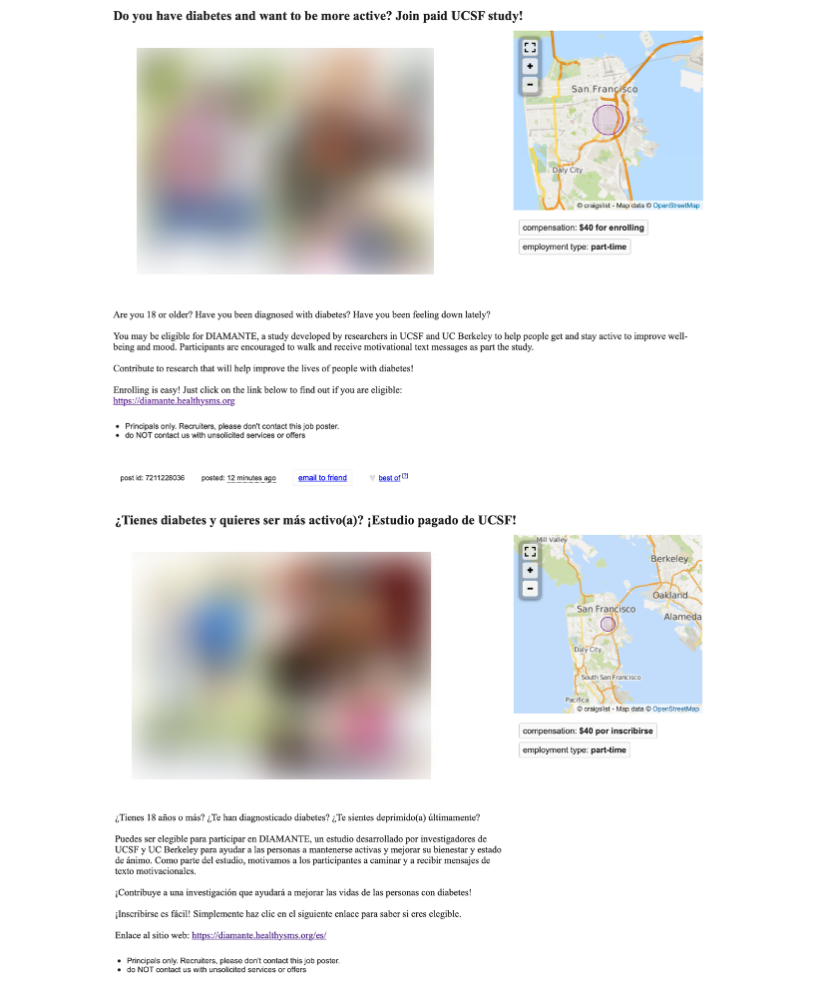
Craigslist posts. DIAMANTE: Diabetes and Mental Health Adaptive Notification Tracking and Evaluation; UC Berkeley: University of California, Berkeley; UCSF: University of California, San Francisco.

We developed Craigslist ad content concurrently with Facebook ads using similar wording and stock photographs. Due to budget constraints and the relatively inflexible and time-consuming ad management interface in Craigslist, we limited this recruitment method to only 2 ad versions, 1 English and 1 Spanish (Figure 3).

### Recruitment

#### Procedures

We recruited US-based individuals using advertisements on Craigslist and Facebook. To identify eligible individuals, we included a Qualtrics (Silver Lake) link on the social media ads to verify inclusion criteria and to determine human identity using a built-in CAPTCHA (Completely Automated Public Turing test to tell Computers and Humans Apart). If eligible, research staff emailed individuals a one-time-use personalized Qualtrics link to a baseline survey. At baseline, participants provided electronic informed consent and filled out a battery of assessments, including demographics and measures of interest. We sent video instructions to participants who completed the baseline survey on downloading the DIAMANTE mobile app based on their mobile operating system (ie, Android or iOS). Upon app download, participants were automatically enrolled into the DIAMANTE text-messaging program. Because the DIAMANTE app monitored how many steps participants took each day, throughout the study, we emailed participants if the app did not capture their steps to address any potential technical difficulties.

#### Facebook

We deployed Facebook ads for approximately 10 weeks (from January 21, 2021, to April 6, 2021). We initially posted 2 English and 2 Spanish ads in the United States with the same set of audience demographics, interests, and education level selected (Textbox 1), except for the ad language (“English [United States]” was set for English ads and “Spanish [All]” for Spanish ads). We saved the English and Spanish demographics sets as reusable “audience” templates for future ads. Based on the selected audience options, Facebook estimated the potential “reaches” for the ads to be 140 million people (English) and 25 million people (Spanish). We set all ads to a daily budget of US $10. Two weeks later, we posted 2 additional English ads. We also activated the “Detailed Targeting Expansion Feature,” which, according to Facebook, “expand(s) your detailed targeting to reach more people when it is likely to improve performance.” After 2 weeks, we discontinued the Spanish ads due to the much higher price to post ads with lower rates of participants completing our study eligibility screener (and no participants enrolled). Five weeks later, we increased the English daily ad budget to US $25 due to a slow recruitment flow. We also shifted to a targeted approach by narrowing the geographical area from the United States to the San Francisco Bay Area and Los Angeles regions.

Textbox 1. **Audience selection for Facebook ads.**

**Demographics and occupation:**

- Cleaning and maintenance
- Construction and extraction
- Healthcare and medical services
- Installation and repair
- Food and restaurants
- Parents (all)
- Ages: 18‐65 years or older
- Genders (all)

**Interests:**

- Community mental health
- Diabetes health
- Mental health awareness month
- Diabetes wellbeing
- Diabetes daily
- Diabetes mellitus II awareness

**Education:**

- Some high school
- High school graduate
- Some college
- Unspecified

#### Craigslist

We deployed Craigslist ads for 32 weeks (from October 9, 2020, to May 20, 2021). Throughout this period, a research team member monitored the ads daily to ensure they were still live (ie, had not expired, been flagged, or removed). Craigslist requires research studies to be posted as paid employment adverts (either in the “Jobs” or “Gigs” sections), so we posted paid ads with a link to the DIAMANTE study landing page (to find out more information about and fill out an eligibility survey for the study) in the “[Location]>Jobs>Et cetera” section. We used a graded approach wherein we started with a set of ads in a specific region, followed by various cities in a specific state, and finally, simultaneous deployment in several cities across multiple states, prioritizing denser areas with large populations of Spanish speakers.

### Data Analysis

We used descriptive statistics to summarize participants’ demographic characteristics for both recruiting methods. For ad performance metrics, we tabulated the duration, total costs, and number of participants recruited through each recruitment platform. We then calculated the cost for enrolled participants for each platform by dividing the total US dollar amount spent in each platform by the number of participants recruited (average cost per participant), number of English speakers (cost per English speaking participant) and number of Spanish speakers (cost per Spanish speaking participant). Cost figures only include direct costs of advertising fees on Facebook and Craigslist; they do not include staff time to develop, implement, and monitor the ads. We compared participant demographics and ad performance metrics between platforms using Pearson chi-square test or Fisher exact tests for categorical variables and one-way analysis of variance for the continuous variable (age). Analyses were carried out on Stata/SE (version 16.1, StataCorp [33].

### Ethical Considerations

The study was approved by the Institutional Review Board Committee at the University of California, San Francisco (IRB: 17‐22608). Participants who screened as eligible provided electronic informed consent in their preferred language, either English or Spanish, were enrolled in the study, and informed of their right to opt out of the study at any time. The demographic data analyzed for this paper are aggregated and thus deidentified. To ensure participant privacy and confidentiality, only institutional review board–approved research staff had access to the data collection platforms. Data were then downloaded from the respective servers and securely stored in a University of California, San Francisco’s Box folder. Participants were not paid for completing the screening survey, but were paid US $40 for completing the baseline questionnaire and US $70 for completing the 6-month follow-up questionnaire.

## Results

### Facebook

Table 1 presents detailed ad performance metrics by platform. Over the 10-week active ads period, our ads were displayed 418,173 times (also known as “impressions”), received 4756 clicks and had a click-through rate of 1.14% (number of impressions ÷ number of clicks), with an average of US $0.44 per click. In the weeks in which both English and Spanish Facebook ads were running, the click-through rate for English ads was 0.98% and the click-through rate for Spanish ads was 2.08%.

**Table 1.** Performance counts by language for Craigslist and Facebook ads (n=76).

Metric	Overall	Facebook	Craigslist	*P* value
Clicked on a study ad and completed study enrollment screener, n	579	71	508	.02
English	507	68	439	
Spanish	72	3	69	
Completed and passed eligibility survey, n	293	58	235	.23
English	263	55	208	
Spanish	30	3	27	
Completed baseline survey and downloaded DIAMANTE app, enrolled in study, n	76	26	50	.29
English	72	26	46	
Spanish	4	0	4	
Ad conversion rate, %	13.12	36.62	9.84	
English	14.20	35.62	10.48	—^a^
Spanish	5.56	0	5.8	

a Not applicable.

Of the individuals who were initially interested from the Facebook ads, 71 went onto the study Qualtrics website and completed study screening (68 from English ads and 3 from Spanish ads). Out of those 71 interested individuals who completed our Qualtrics screening survey, 58 (55 English speakers and 3 Spanish speakers) were found eligible for the study. Out of those 58 eligible participants, 26 (26 English speakers and 0 Spanish speakers) were eventually recruited into the RCT upon completing the baseline survey, downloaded the DIAMANTE app, and randomized into 1 of the 3 study arms. In all, Facebook advertisements recruited 26 participants, averaging US $80.16 per participant (Table 2), with a conversion rate of 36.62%. Notably, we were unable to recruit Spanish speakers through Facebook.

**Table 2.** Recruitment duration, costs, and number of recruits by recruitment platform.

Recruitment platform	Total cost, US $	Campaign duration (days), n	Number of participants recruited, n	Participants per day (number of participants/duration)	Average cost per participant, US $
Facebook	2084.07	76	26	0.34	80.16
English	1703.91	76	26	0.34	80.16
Spanish	380.16	27	0	0	—^a^
Craigslist	7914	224	50	0.22	158.28
English	3110	224	46	0.21	67.61
Spanish	4804	124	4	0.03	1201
Overall total	9998.07	300	76	0.25	131.55
English	4813.91	—	72	—	66.86
Spanish	5184.16	—	4	—	1296.04

a Not applicable.

### Craigslist

We found that it was challenging to determine the effectiveness of recruitment ads in Craigslist for our study because, unlike the more robust ad analytics framework offered by Facebook, Craigslist offers a simple ad management interface for posting content that does not include ad performance metrics such as number of ad views or click-throughs. However, we could track (from participant self-reports within our Qualtrics platform) that 508 individuals (439 English speakers and 69 Spanish speakers) saw an ad-on Craigslist and completed a screener. Of those, 235 (208 English speakers and 27 Spanish speakers) were found eligible for the study after completing the short Qualtrics survey. Out of those 235 eligible participants, 50 were recruited into the study (46 English speakers and 4 Spanish speakers), averaging US $158.28 per participant (US $67.61 for English speakers and US $1201 for Spanish speakers; Table 2). The conversion rate for Craigslist advertisements was, therefore, 9.84% (10.48% for English advertisements and 5.80% for Spanish advertisements). When broken down by language, the recruitment costs varied substantially, with the average cost for English speakers being US $68 and US $1201 for Spanish speakers. This means that the cost to recruit one Spanish speaker was equivalent to recruiting approximately 18 English speakers.

### Participant Characteristics

Table 3 presents the sociodemographic characteristics of the Facebook and Craigslist participants enrolled and randomized in the RCT. Descriptive statistics of respondents across the 2 recruitment platforms revealed similar demographic trends, albeit with some notable differences. The average age (45 years) was the same in both samples. Most respondents were female in both samples (19/26, 73% from Facebook and 32/50, 64% from Craigslist). Craigslist participants tended to be from a higher socioeconomic status than the Facebook participants: a graduate degree (4/26, 15% from Facebook vs 9/50, 18% from Craigslist), currently employed (12/26, 46% from Facebook vs 33/50, 66% from Craigslist), self-reported lower income (16/26, 62% from Facebook vs 23/50, 46% from Craigslist). Race and ethnicity were significantly different between the Craigslist and Facebook samples, with the former being more racially and ethnically diverse than the latter. For example, Whites were overrepresented in the Facebook sample vis-à-vis the general population, with over two-thirds (20/26, 77%) of participants identifying as such. On the other hand, out of 50 Cragslist participants, 18 (36%) identified as White, 14 (28%) as Latino, 8 (16%) as Black, 6 (12%) as Other or Multiracial, and 4 (8%) as Asian or Pacific Islander.

**Table 3.** Participant characteristics (n=76).

Characteristic	Overall(n=76)	Facebook(n=26)	Craigslist(n=50)	*P* value
Age (years), mean (SD), range	45 (11.74), 24-68	45 (13.24), 26‐68	45 (11.02), 24‐67	.83
Gender, n (%)				.50
Male	23 (30)	6 (23)	17 (34)	
Female	51 (67)	19 (73)	32 (64)	
Other	2 (3)	1 (4)	1 (2)	
Language n (%)				.29
English	72 (95)	26 (100)	46 (92)	
Spanish	4 (5)	0 (0)	4 (8)	
Education, n (%)				.72
Some high school or less	4 (5)	0 (0)	4 (8)	
High school graduate or general educational development	10 (13)	4 (15)	6 (12)	
Some college or tech school	20 (26)	7 (27)	13 (26)	
College graduate	29 (38)	11 (42)	18 (36)	
Graduate degree	13 (17)	4 (15)	9 (18)	
Race and ethnicity, n (%)				.01
White or Caucasian	38 (50)	20 (77)	18 (36)	
Black or African American	11 (14)	3 (12)	8 (16)	
Asian or Pacific Islander	4 (5)	0 (0)	4 (8)	
Latino or Hispanic	15 (20)	1 (4)	14 (28)	
Other or multiracial	8 (11)	2 (8)	6 (12)	
Employment, n (%)				.16
Full time	32 (42)	11 (42)	21 (42)	
Part time	13 (17)	1 (4)	12 (24)	
Homemaker	6 (8)	3 (12)	3 (6)	
Unemployed	13 (17)	5 (19)	8 (16)	
Disabled or on disability	8 (11)	5 (19)	3 (6)	
Retired	3 (4)	1 (4)	2 (4)	
Other	1 (1)	0 (0)	1 (2)	
Low income, n (%)^a^				.20
Yes	39 (51)	16 (62)	23 (46)	
No	37 (49)	10 (38)	27 (54)	

a We labeled responses to the subjective income ladder question as: 1‐5 for low income and 6‐10 for not low income.

## Discussion

### Principal Findings

Our results support the feasibility of recruiting diverse respondents for a digital health trial through social media. While Craigslist study recruitment ads reached more people, resulting in more completed screening surveys than Facebook ads, there was a higher number of ineligible and incomplete enrollment from Craigslist compared with Facebook. Yet, despite the overall ad conversion rate being higher on Facebook than on Craigslist, Facebook ads proved more expensive (when only accounting for English speaking participants) and, unlike previous recruiting outcomes by Bunge et al [17], ineffective for recruiting Spanish speakers. Importantly, participants recruited through Craigslist were more ethnically and racially diverse than those recruited from Facebook. We did manage to recruit a small number of Spanish speakers through Craigslist; this finding aligns with the work by Pratap et al [8], which found this platform to be more effective in recruiting Spanish speakers than targeted advertising through Facebook and Twitter. However, participants recruited through Craigslist only accounted for 5.3% (4/76) of our total sample, even though we intended to have at least a sample of Spanish speakers proportionate to that found in our prepandemic clinical sample recruited through traditional methods (Spanish speakers accounted for 67% (10/15 of our clinical sample). With the cost of recruiting Spanish speakers being substantially higher than English speakers, even more than that reported in previous literature, we confirm the need for substantial financial resources to be injected into digital recruitment efforts in order to sample from this marginalized population more effectively. Budgets of studies that seek to include this group should consider this premium expense. It also appears that an online-only recruitment strategy alone may be ineffective for Spanish speakers. Other mHealth studies have successfully recruited Spanish speakers by coordinating with community organizations to develop trust [34], indicating the potential merit of applying a variety of approaches to recruit hard-to-reach populations in such studies [35].

This study had some limitations. First of all, because we had limited resources yet wanted to create engaging advertisements for Craigslist and Facebook users to recruit from for our study sample in a short amount of time, we conceptualized the ads themselves as a “product to design for” under the UCD framework without stakeholder input. Ideally, we would have asked for feedback directly from targeted audiences in developing such ads, but we were constrained logistically and financially during the COVID-19 pandemic when the online recruitment for this study launched. Co-design with stakeholders strategy could have enhanced the ads’ effectiveness, particularly when finding the right balance of advertising specificity (to target specific groups like low-income Spanish speakers) versus reach (to attain a representative sample). For example, although we produced many ads using personas, we ultimately selected a small number of such ads; not including input from end users, particularly of Spanish speakers, in this selection process is a clear limitation. Another limitation is that the timeframe and money spent in both campaigns differed. For example, the fact that the Facebook ads were futile in recruiting Spanish speakers could be due to the shorter campaign duration compared with the Craigslist campaign duration. Our sense, however, was that the Spanish ads on Facebook were simply ineffective (ie, the price post them was very high despite the low rates of participants completing the eligibility screener), regardless of the timeframe, given the much lower engagement they generated among the target population versus the English ads. Furthermore, our cost calculations are not comprehensive, as we did not factor in staff time to develop, implement, and monitor the ads in our cost calculations, beyond the advertising fees charged by the social media platforms. Although this is not inconsistent with cost reporting found in relevant previous literature [8,17], future studies should carefully record and account for staff implementation costs in order to generate more precise cost-effectiveness results of social media study recruitment ads. In general, having a more consistent recruitment, enrollment, and cost reporting standards would allow for more data comparability across mHealth studies [35,36]. Finally, we found it difficult to draw upon best practices in the mHealth literature for recruitment procedures through social media; while there are guides on the use of social media for health research recruitment [37], most studies provide minimal details of their social media recruitment protocols and strategies. Thus, to advance the top public health imperatives of mitigating health service inequities, the field of digital health must adopt more rigorous reporting standards and data transparency of recruitment methods.

Our study proved that it is challenging, albeit possible, to recruit individuals from marginalized groups for digital health trials through social media channels. This methodology allowed us to recruit a diverse sample in terms of socioeconomic and racial characteristics. However, the low number of recruited Spanish speakers implies that this population may have additional recruitment barriers to pursue. Understanding the best methods for effectively recruiting such a hard-to-reach group is crucial for advancing digital health science. Future research should directly ask targeted audiences about barriers and facilitators to enrolling in a study online to help address the challenges by modifying recruitment procedures. Trust may be a critical factor in recruiting Spanish speakers who may be wary of consenting online without having a previous relationship with researchers, especially for individuals who started or completed the study screening but did not ultimately enroll in the trial. Indeed, mHealth studies with other marginalized groups have reported higher outreach outcomes of eligible participants who identified as a racial or ethnic minority through in-person methods as opposed to social media advertising [22].

It may also be worthwhile to test further whether other online methods, beyond paid advertisements, are needed to recruit diverse samples. Using posts and other paid and free methods on Facebook (ie, pages, sending friend invites to prospective participants, and untargeted ads) has been found effective in previous studies [10,36]. Furthermore, content-sharing platforms such as YouTube (Google), TikTok (ByteDance), and Instagram (Meta) may have something beneficial to offer in the targeted recruitment of individuals belonging to marginalized communities for research purposes, particularly when it comes to the use of video. Future studies should continue to test ad customization and content development to advance the research community’s understanding of best practices on the recruitment of diverse populations.

Finally, it will be important to continue to report data from online recruitment strategies to refine methods and approaches as technology evolves. This is especially true as we seek to confirm participant identity (using CAPTCHA and other methods to remove bots), given that the large number of participants from Craigslist that did not convert into the study could have been linked to computer-generated responses to our social media ads.

### Conclusion

Results from this study revealed that it is possible to recruit diverse sample sets using social media advertisements. However, despite targeted recruitment efforts, social media recruitment of Spanish speakers proved especially challenging and costly. Further research is needed to determine systematic, online methods for recruiting marginalized communities into digital health studies to advance the public health goal of reducing service inequities. Recruiting samples that reflect the racial and ethnic diversity of the US population in rigorous studies assessing mobile mental health interventions, particularly RCTs, is thus crucial for the mHealth promise of ameliorating racial and ethnic service inequities to unfold as intended.

## Supplementary material

10.2196/56329 Multimedia Appendix 1 Advertisement copy by persona.

## Abbreviations

CAPTCHA: Completely Automated Public Turing test to tell Computers and Humans Apart
COM-B: Capability, Opportunity, Motivation and Behavior
DIAMANTE: Diabetes and Mental Health Adaptive Notification Tracking and Evaluation
mHealth: mobile health
RCT: randomized controlled trial
