# Supplementary material for: Using Social Media to Recruit a Diverse Sample of Participants for a Mobile Health (mHealth) Intervention to Increase Physical Activity: Exploratory Study
Source: JMIR Mhealth Uhealth. 2025 Apr 28;13:e56329. doi: 10.2196/56329 (PMC12052219; doi:10.2196/56329)
Supplement: Multimedia Appendix 1 [file mhealth-v13-e56329-s001.docx]

*Ads copy by persona*

|  | Persona | Benefit | Self-efficacy | Opportunity |
| --- | --- | --- | --- | --- |
| 1a | English Broad Health Conditions | Diabetes can affect mood and create feelings of stress. Staying active can help.    Diabetes Research - The DIAMANTE project aims to help people stay active with daily motivational texts | People with diabetes can live happy and healthy lives      A UCSF project for people with diabetes to improve mood and wellbeing | **Can text messages help people with diabetes live healthier lives?**    **UCSF study to get daily tips on how to stay active** |
| 1b | English Broad Wellbeing | **Getting active can help with low mood and feelings of stress.**    **Wellbeing Research - The DIAMANTE project aims to help people get and stay active with daily motivational texts** | Anyone can live a happy and healthy life    A UCSF project to improve mood and wellbeing through physical activity | Can text messages help people live healthier lives?    UCSF study to get daily tips on how to stay active |
| 2 | English speaker, white, female, younger | Exercise can help people with diabetes manage their symptoms and improve wellbeing    UCSF texting study for exercise and diabetes management | Improving physical and mental wellbeing is possible    UCSF texting study to receive tips on prioritizing health | It’s possible to keep the mind and body active while staying at home!    Daily text messages on how to take care of wellbeing for people with diabetes |
| 3 | English speaker, white, male, older | Diabetes can create feelings of stress and affect mood    Text messaging trial for people with diabetes | How to get back on track and achieve physical activity goals?    UCSF trial for people with diabetes: supportive texts messages to get more active | How can people with diabetes maximize their physical and mental health?    UCSF text-messaging study to get more active and start feeling better |
| 4 | English speaker, Hispanic/Latino, male, older | Being active is important for health, and our health is important for our loved ones    UCSF study for people with diabetes | Staying healthy for our family and community    Mood and diabetes text-messaging support available | How not to let pain get the best of us?    Join UCSF study and get daily messages to improve health |
| 5 | English speaker, Latina, female, older | Our loved ones want us to be healthy and exercise can help us get there    UCSF study for people with diabetes to get more active and improve mood | Staying active is hard but not impossible    UCSF text-messaging study to manage mood and diabetes | **Support available for people with diabetes and depression**    **Study to receive tips and motivational messages to be more active and cope with physical pain** |
| 6 | English speaker, Black, male, older (1) | **Having diabetes and low mood can lead to burnout**    **Prioritize your health and wellbeing by enrolling in a UCSF study** | Living happy and healthy with diabetes is possible    Join UCSF study to receive tips on how to be more active and improve mood | Finding it hard to stay motivated to be active?    Join a UCSF wellbeing study to help you stay on track |
| 7 | English speaker, Black, female, older (1) | People with diabetes sometimes get down. Getting active can help.    Join texting study to receive help with getting active and feeling better | **Times are tough right now but taking control of health is still possible**    **Join UCSF texting study to increase wellbeing** | Worried about health? Losing hope that things will get better?    Text messaging support for people with diabetes and low mood |
| 8 | English speaker, Black, male, older (2) | It is important to stay positive and motivated to exercise, even during tough times    Text messaging support for people living with sugar and low mood | People living with sugar can take control of their health by staying active    Texting study to manage walking routine and feel better | Struggling to stick to a walking routine?    Get tips on staying active by joining a texting study for people living with sugar and low mood |
| 9 | English speaker, Black, female, older (2) | Taking care of health is not just important for us -- it is important for our families too    Text messaging support available for people with diabetes and low mood | Living with sugar is hard but staying positive and active can help    Text messaging support available to stay active and improve mood | Living with diabetes can take a toll on mood and wellbeing    Texting support from a UCSF study to stay active and feel better |
| 10 | English speaker, Asian, female, older | Staying active can help people with diabetes live a long life!    UCSF text-messaging study for improving mood and staying on track | Staying healthy for ourselves and our loved ones is possible    A UCSF wellbeing project for people with diabetes and low mood | Feeling low? Finding it hard to get much walking done?    UCSF text-messaging study to help keep people with diabetes and low mood stay on track |
| 11 | English speaker, Asian, male, older | Diabetes can create feelings of stress and affect mood    Text messaging study for people with diabetes | Whether we do it for ourselves or our loved ones, achieving our exercise goals is possible!!    UCSF study for people with diabetes and low mood: supportive text messages to get more active | How can people with low mood and diabetes maximize their wellbeing?    Join a UCSF text-messaging study to stay active and prioritize health |
| 12a | Spanish Broad Health Conditions | La diabetes puede afectar el estado de ánimo y crear sentimientos de estrés. Mantenernos activos puede ayudar.    Investigación sobre la diabetes - El proyecto DIAMANTE tiene como objetivo ayudar a las personas a mantenerse activas con mensajes de texto diarios | **Las personas con diabetes pueden llevar una vida feliz y saludable**    **Un proyecto de UCSF para gente con diabetes que quieren mejorar su estado de ánimo y bienestar** | ¿Pueden los mensajes de texto ayudar a la gente con diabetes a llevar una vida más saludable?    Estudio de UCSF para obtener consejos diarios sobre cómo mantenernos activos |
| 12b | Spanish Broad Wellbeing | Estar activo puede ayudar con el mal humor y los sentimientos de estrés    Investigación sobre el bienestar - El proyecto DIAMANTE tiene como objetivo ayudar a mantenernos activos con mensajes de texto diarios | Todos podemos vivir una vida feliz y saludable    Proyecto de UCSF para mejorar el estado de ánimo y el bienestar a través de la actividad física | **¿Pueden los mensajes de texto ayudar a la gente a llevar una vida más saludable?**    **Estudio de UCSF para obtener consejos diarios sobre cómo mantenernos activos** |
| 13 | Spanish speaker, Hispanic/Latino, male, older (1) | La diabetes puede crear sentimientos de estrés y afectar el estado de ánimo    Estudio de mensajes de texto para personas con diabetes | ¿Cómo podemos alcanzar nuestras metas de actividad física?    Estudio de UCSF para gente con diabetes: mensajes de texto de apoyo para ser más activos | ¿Cómo puede la gente con diabetes optimizar su salud física y mental?    Estudio de mensajes de texto de UCSF para ser más activos y sentirse mejor |
| 14 | Spanish speaker, Hispanic/Latina, female, older (1) | La gente con diabetes a veces se deprime. El ejercicio puede ayudar.    Estudio de mensajes de texto para recibir ayuda para mantenerse activa y sentirse mejor | Estos son tiempos difíciles, pero tomar el control de nuestra salud es posible    Únase al estudio de mensajes de texto de UCSF para mejorar su bienestar y estado de ánimo | ¿Preocupada? ¿Perdiendo la esperanza de que las cosas van a mejorar?    Programa de mensajes de texto para apoyar a las personas con diabetes y bajo estado de ánimo |
| 15 | Spanish speaker, Hispanic/Latino, male, younger | El ejercicio puede ayudar a la gente con diabetes a controlar sus síntomas y mejorar su bienestar    Estudio de mensajes de texto de UCSF para motivarse a hacer ejercicio y controlar la diabetes | Es posible mejorar nuestro bienestar físico y mental    Estudio de mensajes de texto UCSF para recibir consejos sobre cómo llevar un control de la diabetes y mejorar el estado de ánimo | ¡Es posible mantener la mente y el cuerpo activos estando en casa!    Mensajes de texto diarios sobre cómo mantenerse activo y sanos para la gente con diabetes y estado ánimo bajo |
| 16 | Spanish speaker, Hispanic/Latino, male, older (2) | Tener diabetes y bajo ánimo puede llegar a ser difícil    Estudio de UCSF para recibir mensajes de texto para motivarse a estar activo | Vivir feliz y saludable con la diabetes es posible    Únase a un estudio de UCSF para recibir consejos sobre cómo ser más activo y mejorar el estado de ánimo | ¿Le cuesta mantenerse motivado para ser activo?    Únase a un estudio de UCSF para ayudarle a mantenerse en buen ánimo y controlar la diabetes |
| 17 | Spanish speaker, Hispanic/Latino, female, younger | La diabetes puede crear sentimientos de estrés y afectar el estado de ánimo    Estudio de mensajes de texto para gente con diabetes | Es posible lograr nuestras metas de ejercicio.    Estudio de UCSF para gente con diabetes y bajo estado de ánimo: mensajes de texto de apoyo para hacer actividad física | La salud es lo primero - permanece activa y mejora tu bienestar    Únete a un estudio de mensajes de texto de UCSF para gente con diabetes y bajo ánimo |
| 18 | Spanish speaker, Hispanic/Latino, female, older (2) | Cuidar de la salud no es sólo importante para nosotros... es importante para nuestras familias también.    Mensajes de texto de apoyo disponibles para personas con diabetes y bajo estado de ánimo | Vivir con diabetes es difícil, pero mantenerse positiva y activa puede ayudar    Mensajes de texto de apoyo disponibles para mantenerse activa y mejorar el estado de ánimo | Vivir con diabetes puede afectar el estado de ánimo y el bienestar...    Mensajes de texto de un estudio de UCSF para mantenerse activa y sentirse mejor |
| 19 | Spanish speaker, Hispanic/Latino, female, older (3) | El ejercicio puede ayudar a las personas con diabetes a vivir una larga vida    Estudio de mensajes de texto de UCSF para mejorar el estado de ánimo y tener motivación para hacer ejercicio | Mantenernos sanos para nuestros seres queridos es posible    Proyecto de bienestar de UCSF para gente con diabetes y estado de ánimo bajo | ¿No tiene motivación? ¿Le cuesta mucho caminar?    Estudio de mensajes de texto de UCSF para ayudar a las personas con diabetes y bajo estado de ánimo a mantenerse motivadas para hacer ejercicio |
| 20 | Spanish speaker, Hispanic/Latino, male, older (3) | Es importante mantenerse positivo y motivado para hacer ejercicio, incluso en tiempos difíciles.    Mensajes de texto de apoyo para la gente que vive con diabetes y ánimo bajo | Las personas que viven con diabetes pueden controlar su salud manteniéndose activas    Estudio de mensajes de texto para manejar la rutina de caminar y sentirse mejor | ¿Le cuesta trabajo mantener la rutina de caminar?    Consejos para mantenerse activo: estudio de mensajes de texto para personas que viven con diabetes y bajo estado de  ánimo |

Note: Bold text indicates the ads that were actually used in Facebook recruitment.
